# Trust and mistrust in fan relationship management: survey evidence on Russian football fans

**DOI:** 10.3389/fspor.2025.1715122

**Published:** 2025-12-05

**Authors:** Kristoff Reichel, Ilia Sannikov, Christian Brandt, Markus Kurscheidt

**Affiliations:** Department of Sport Governance and Event Management, BaySpo – Bayreuth Center of Sport Science, University of Bayreuth, Bayreuth, Germany

**Keywords:** trust and mistrust, fan relationship management, Russian football, Fan ID, football governance, sport governing bodies, supporters

## Abstract

**Introduction:**

Trust is a key factor in governance and business. This is notably the case in professional sports with the large stakeholder group of fans as they claim a more active role than consumers in other industries. Thus, they may influence sport policy directly or indirectly, positively or negatively, through their communication, self-organization, coordinated activism and behavior. Particularly in football, the relationship between fans and sport organizations has attracted the interest of researchers. However, empirical studies primarily focused on Western football leagues within liberal political and cultural environments. Trust and mistrust in institutions may be more important in state-regulated environments, such as in the Russian Federation. Therefore, this study investigates the level of trust and questions the formation of trust in sport governing bodies among Russian football fans.

**Methods:**

This article presents evidence from the first large-scale survey of supporters of the Russian Premier League (*N* = 4,090) with a focus on fans' attitudes, behaviors, and concerns. It is based on a questionnaire covering relevant issues in football governance that had been applied in different environments before. Adapted to specificities of Russian football, in particular, an item battery on the construct of trust was added: “How much do you trust the following institutions with regard to the organization of football?” The survey was distributed online in September and October 2022 via more than fifteen Telegram channels. Due to the wide regional distribution of participants and high sample size, the dataset is considered approximately representative of active Russian football supporters who engage on social media. Ordered logit regressions of trust variables were performed on numerous explanatory variables of attitudes, behaviors, and sociodemographics.

**Results:**

The findings reveal that supporters express the greatest confidence in supporter organizations, followed by clubs, while trust in local football organizations, such as the league and the association, as well as in international bodies including UEFA and FIFA, tends to be more limited. The regression models on trust variables regarding the favorite club and the league governance confirm that good relations with the fans, all else equal, foster trust in the football organizations while disrespect for the needs and self-concept of fans lower trust. In contrast to Western European fans, Russian supporters value elements of commercialization as modernization and respond with higher trust levels. Yet, closer emotional attachment to their club makes fans more skeptical about the trustworthiness of football officials while the belief in reforms of Russian football strengthens trust in the governing bodies.

**Discussion:**

The findings and insights from the survey clearly emphasize that clubs and league governing bodies should acknowledge fans' active role distinguishing them from ordinary customers. If football officials wish to strengthen fans' trust they should invest in fan relations. This is in line with evidence on Western European fans. However, the results in detail show distinct differences which underlines the need for more diverse evidence on fan attitudes worldwide. The institutional environments matter and deserve more attention in sports economics and management research.

## 1 Introduction

Trust has increasingly attracted attention in sports-related research as a key factor in governance and economics. It is a multidimensional phenomenon that is often conceptualized in contrast to control. To trust another party means to accept a degree of vulnerability by relinquishing control (1). Trust has been described as the “essence of collaboration” (2), enabling deeper forms of cooperation, reducing complexity (3), and lowering the transaction costs associated with monitoring and controlling others. By contrast, distrust entails negative perceptions and expectations, coupled with an unwanted vulnerability (4). Distrust often provokes intensive monitoring and control, generating additional costs. Consequently, the dynamics of both trust and distrust are highly relevant to sport management and governance.

Trust is relevant for organizational [e.g., (1)] and systemic governance, and therefore, involves large networks of stakeholders (5). In football, one of the most popular sports in the world, fans are a very important stakeholder, especially for clubs and associations, as they are not only consumers of professional sport, but can also influence sports policy directly or indirectly, positively or negatively, through their behavior. Yet, research consistently shows that fans often express distrust toward governing bodies (6, 7). They often accuse federations and associations of privileging commercial, media, or political interests over the concerns of supporters. Fans express their distrust towards FIFA, for example. They often perceived the organization as self-interested and untrustworthy, even comparing it to a mafia-like structure, which led to repeated fan protests (8).

A second area that generates distrust among fans concerns security measures. Fans often fear what they describe as repression (9). While associations, politicians, and police forces proclaim their intention to prevent violence, they remain skeptical of fans' assurances of peaceful behavior. Conversely, fans express distrust toward these institutions, anticipating restrictions on civil rights, repressive practices, and the suppression of critical voices directed at clubs or football more broadly. Protests against identification systems, such as the implementation of Fan ID cards in the United Kingdom (10), Italy (11), and Turkey (12), as well as mandatory passport checks in the Czech Republic (10) and Ukraine (13), illustrate these tensions. In each of these cases, federations planned to sell matchday tickets only to registered fans who could be easily identified. Such practices of distrust generate substantial costs not only for fans but also for associations, authorities, and the sport as a whole.

Given this significance, Garcia and Llopis-Goig (14) studied the extent to which fans of European football clubs trust different governing bodies. Their results show the highest levels of trust in fan organizations and the lowest in national governments. Additionally, they developed a taxonomy, consisting of five types with different preferences on trust and distrust in club owners, federations, politics, or supporter organizations. However, with most empirical work on sport governance, these studies concentrated on European sports leagues. But research shows that phenomena such as commercialization are perceived differently in other cultures elsewhere, and that fans' perspectives therefore differ from those in Europe (15, 16). Furthermore, it is essential to expand the existing literature and to include sports cultures operating in strongly authoritarian environments, where politics exerts a profound influence on sport and its sociocultural meanings (17). Therefore, this article responds to that need by investigating trust among football fans in Russia, a non-Western country that e.g., implemented a Fan ID card in 2022 within a highly centralized political system.

The approach is guided by the following two research questions:

1. What are the key factors that influence trust and mistrust in fan relationship management among Russian football fans, as evidenced by survey data?
2. What is the level of trust among Russian football fans in the club governance as well as national and international sport governing bodies?

These results are compared with those of the European surveys, to elaborate both the particularities and the similarities of fans and trust on a global scale.

The structure of the article is as follows. The next section examines the theoretical foundations of trust and its relevance for governance. It then considers trust in the context of sport, with a particular focus on football, and outlines the Russian case, paying special attention to the introduction of the Fan ID system. The methodology section presents the survey design, data collection, and analytical approach. The subsequent section reports the empirical findings and compares them with European evidence. The article concludes by discussing theoretical and practical implications and identifying directions for future research.

## 2 Literature review

### 2.1 Theoretical aspects of trust

In recent years, research on trust has expanded considerably across different disciplines, underscoring its relevance (18, 19). Trust serves as a framework for structuring both interpersonal and institutional interactions. Multiple definitions have been proposed, each describing different spheres of humanity (20). Lewicki et al. (21) define trust as “confident positive expectations regarding another's conduct” (21), whereas Blomqvist (22) emphasizes its reliance on the predictable behavior of institutions. These relations are described as: trust, mistrust, and distrust. Trust is associated with attitudes such as “loyalty, commitment, and confidence”, which in turn foster “compliance, sympathetic judgment, and participation”. Mistrust reflects a more negative relation related to “caution, watchful, questioning”, resulting in “making effort to be informed, alert, on standby to act”. Distrust, by contrast, is linked to “insecurity, cynicism, contempt, fear, anger, alienation”, which may lead to “withdrawal, defiance, support for populist challenge or empowerment movement” (23).

From a psychological perspective, trust is shaped by shared characteristics, reputation, behavior, and authority, all of which influence people's individual cognitive biases (24). For the purposes of this study, the focus is placed on trust in public institutions, while interpersonal trust or trust within organizations is considered less directly (25). Zucker (26) distinguishes three sources of institutional trust. Process-based trust arises from previous experiences people have had with an institution, whether direct or reported by others. Characteristic-based trust refers to confidence generated by general features of the institution, such as the fact that a board is democratically elected, even without personal experience of its performance. Institutional-based trust is rooted in cultural norms, traditions, and symbolic legacies, including predispositions to trust or distrust (24). However, our study does not distinguish different ways of gaining trust; it rather investigates the status quo of trust. Trust is widely regarded as essential for social cooperation (24), it plays an integral role in governance of complex systems, including contemporary states (27, 28). Institutional trust also has a rational foundation, shaped by performance: institutions perceived as well-functioning generate trust, while untrustworthy ones provoke skepticism and distrust (29).

Existing literature identifies several socio-economic determinants of trust in public institutions. Income is frequently highlighted. Higher income has a positive effect on trust in state institutions (30, 31), including the police (32). By contrast, lower income and lower education (33), along with social inequality and exclusion, contribute to a climate of distrust in state institutions, including the police (34). Developments of trust do not necessarily need to be economically rational based. A study of Romashkina et al. (35) on developments on trust in Russia state authorities between 2006 and 2016 show, that trust increased during that period, while the standard of living decreased. The political culture, transparency or cultural sensitivity in relation to minorities are influencing the level of trust (34).

### 2.2 Trust in the context of sports

Within the domain of sport, trust has become an increasingly important determinant of organizational legitimacy and sustainability. It is widely regarded as a strategic value that underpins the effective functioning of sport organizations and their governing bodies (36). Conversely, the absence of trust has had visible consequences. Failed referendums on Olympic bids in several Western countries have been attributed in part to public skepticism toward the International Olympic Committee (IOC), its governing bodies and related organizations (37–39).

Football provides particularly clear evidence of how trust shapes the relationship between supporters and governing bodies. In the context of fans and supporters, the literature suggests that good governance structures can provide trust and legitimacy (40). Yet, despite these possibilities, distrust remains widespread, especially towards national governing bodies, club owners, and presidents' clubs (14). Addressing this problem is essential for organizational sustainability. Sport organizations must recognize the long-term value of supporters and adopt effective communication strategies, since the loss of trust can lead to alienation, declining attendance, and negative behavioral intentions (41–43).

Recent studies offer further insight into these dynamics. Research on Japanese elite sport organizations (25), related to the Tokyo Olympics demonstrated a marked decline of trust in institutions between 2013 and 2021. The National Olympic Committee was evaluated lowest (3.67 out of 10), while other institutions did not score much higher. The authors attribute this decline in trust to the scandals that preceded the Tokyo Olympics. Interestingly, the same study also found that people who favored tradition and authoritarian politics tended to express higher trust in elite sport institutions (25), suggesting that broader political orientations shape institutional credibility.

Qualitative research provides concrete illustrations of distrust expressed by fans towards football's national and international governance bodies. Such expressions appear in murals (8), banners (44) or statements (13, 45). This distrust is also reflected in fans' desire for greater involvement in football governance (46). Quantitative findings complement these accounts. A recent study on Belgium football fans, for instance, revealed lower levels of trust in the integrity of football compared to other sports (47). More broadly, fans frequently report the perception that organizations and players prioritize financial gain over genuine concern for supporters (48).

In this context, this paper aims to determine supporters' perceptions of trust in governing bodies locally and internationally in Russia by comparing Garcia's and Llopis-Goig's (14, 40) results in Europe (France, Germany, Poland, Spain, Turkey and United Kingdom). Their survey asked supporters to evaluate their trust or distrust in club presidents/owners, national sport governance bodies, international football governing bodies, control agents and supporter organizations. Results indicate that fans place the greatest trust (measured with a five-point Likert scale) in fan groups (*M* = 3.48, *SD* = 1.05) and club management (*M* = 3.23, *SD* = 1.05). However, their skepticism increases as the distance from the object of affiliation grows. FIFA received the lowest mean score (*M* = 2.31, *SD* = 1.23), and the national governments were also rated poorly (*M* = 2.14, *SD* = 1.09). However, cross-national differences between fans were also evident. Polish fans reported the highest trust in fan groups (*M* = 3.91), followed by France fans (*M* = 3.07). In Spain, trust in club management was lower than in other countries (*M* = 2.36). Moreover, Polish fans also tend to express the highest trust in the professional league (*M* = 3.25), whereas Spanish (*M* = 1.96) and especially Turkish fans (*M* = 1.79) expressed strong distrust in that organization. Consistently, similar patterns emerged in evaluations of national federations and governments, which were most mistrusted in Spain and Turkey. Interestingly, Polish fans expressed relatively high trust in FIFA (*M* = 3.40), while in the United Kingdom (*M* = 1.87) and Germany (*M* = 1.85), FIFA was the least trusted body. Beyond cross-national variation, sociodemographic differences added further nuance. Male respondents generally consistently expressed lower trust in governance bodies than female respondents, across clubs (owners/presidents), national organizations, and international federations. As respondents grow older, their outlook becomes more skeptical.

In relation to national sport governance bodies, the highest mistrust appeared among fans aged 21–30, whereas younger supporters below this age (especially 15 and 16) and older fans over 50 expressed higher trust. Moreover, trust in international governing bodies declines with increasing fan age. These results provide a framework for the present study. We compare them with trust in the Russian football context, which is shaped by distinctive institutional and political conditions. Specifically, the study assesses Russian supporters' perceptions of trust in football governing bodies at both the local and international levels and contrasts them with the findings reported by Garcia and Llopis-Goig (14, 40) in Europe for France, Germany, Poland, Spain, Turkey, and the United Kingdom.

### 2.3 Trust in the Russian context: fans against the system

The dynamics of trust acquire particular significance in Russia, where cultural, historical, and political factors have shaped distinctive patterns of governance and public perception. The centralized governance system, formed and survived in the Soviet Union, continues to shape public perception in contemporary Russia.

This has been reflected in the sporting environment, where football has long been not only a game but also an instrument of state policy. Russia's football model differs from those of the European counterparts, as it has relied heavily on federal state- and state-owned enterprises (49). Football and its institutions are therefore closely linked to political elites and embedded within the broader state-capitalist economic system (35) more directly than in Western European countries. Under such conditions, the role of the fans has always extended beyond the traditional support of the team. Fans have frequently been drawn directly or indirectly into political processes, engaging in activism, protests, and social movements, and the establishment of collective identities aimed at achieving deeper social change through the sport (50). Since previous studies have not directly examined trust in Russian football governance, this study specifically addresses the perspective of fans. The timing was chosen accordingly, as in 2022, the Russian authorities implemented fan ID card for all spectators attending Russian Premier League matches. It underlined the skepticism of state authorities towards fans, which in turn has caused widespread mistrust among fans. The system provides state institutions with the ability to identify and monitor individuals attending matches and, if deemed necessary, to restrict access without judicial oversight. Such introductions have already led to fan protests in several European countries like Italy (11), Turkey (12), and Croatia (51) as well as beyond, e.g., Mexico (52).

The preconditions for implementation in the Russian context might be difficult relations between fans and law enforcement agencies. While fans often express concerns about policing methods, law enforcement emphasizes the need to enhance security measures (53). Yagodin (53) has described this dynamic as a form of “cold war.” Certain parallels can be drawn between the occasionally stringent actions of law enforcement toward fan groups and the broader security practice observed during public demonstrations in recent years (54). However, it is essential to acknowledge that elements of far-right ideology persist within segments of the fan community, reflected in racist provocations at matches, interethnic clashes, and conflicts (55). Consequently, football fans have often been regarded by law enforcement as a constituency requiring particular monitoring and preventive attention (56).

After the implementation of the fan ID card in Russia, resistance was immediate and widespread. Several months before the law took effect, fans of 15 out of 16 Russian Premier League clubs organized a boycott (57), a collective response that can be interpreted as a behavioral response to distrust (23). This case was particularly notable because, despite the disagreements and long-standing rivalries, fans of different clubs were able to unite in defense of their rights (54). The protests were acknowledged at the highest political level, with the president's press secretary encouraging clubs to engage in dialogue with supporters (58). Officials and club management sought to reach a compromise with the fans; however, these efforts did not succeed, and the law was ultimately implemented.

The consequences were visible soon after implementation. Attendance declined markedly during the first half of the 2022/23 season. FC Rostov, for example, which had previously attracted around 35,000 spectators to home matches, recorded attendances of about 5,000 following the introduction of fan ID card (59). The measure also contributed to a noticeable transformation in the composition of stadium audiences. In particular, the share of women and children increased, suggesting a shift in the social profile of attendees and raising questions about the broader cultural implications of the fan ID policy (60). This example illustrates how governance arrangements influence fan institution relations in contemporary football.

Against this background, the present study examines the level of trust among Russian football fans and compares the findings with those of Garcia and Llopis-Goig's European survey.

## 3 Methodology

### 3.1 Research design and measurement

This empirical study investigates supporter behavior and attitudes, with a specific focus on how Russian football fans perceive current governance structures and the level of trust in professional football in Russia. A questionnaire was selected as the most appropriate research instrument due to its ability to capture a wide and diverse range of opinions, attitudes, and preferences (61).

The questionnaire was developed based on established fan research, including studies on psychological attachment to teams (6, 62), and broader debates on football governance (46, 63). For comparative purposes with similar work on Chinese football fans (15), the instrument was adapted to reflect the unique aspects of Russian football, such as the implementation of fan ID policies and the exclusion of Russian teams from international competitions due to the military conflict in Ukraine.

### 3.2 Questionnaire structure and variables

The questionnaire is structured into the following key categories: (1) self-reported attendance behavior, (2) membership or attachment, (3) fan identity, (4) attitudes toward commercial issues in football, (5) attitudes toward football governance, (6) attitudes toward club governance, (7) behavioral intentions (regarding future fan behavior), and (8) sociodemographic data. As illustrated in Table 1, the dependent variables TRUST_CLUB (mean of two variables) and TRUST_RUFB (mean of three variables) capture general attitudes toward fan trust.

**Table 1:** Overview of the item and factor variables.

Variable	*N*	*Values*	*M*	*SD*	Description
Dependent variables					
TRUST_CLUB	3,090	5	3.23	0.98	Trust in Russian club governance (mean of 2 variables) (1‒5 sc.)
BINTRUST_CLUB	2,879	2	0.80	0.40	Binary of TRUST_CLUB [1 = (somewhat) agree]
TRUST_RUFB	3,090	5	2.43	0.98	Trust in Russian football governance (mean of 3 variables) (1‒5 sc.)
BINTRUST_RUFB	2,558	2	0.47	0.50	Binary of TRUST_RUFB [1 = (somewhat) agree]
Attendance behavior (self-reported) and season ticket holders					
ATTENDANCE*	3,781	30	4.16	4.01	Attend. pref. (mean of HOME & AWAY)
HOME	3,781	17	5.17	5.33	Home attendance per season (metric)
AWAY	3,781	16	3.15	4.55	Away attendance per season (metric)
SEASON	3,781	2	0.10	0.30	Season ticket holder (1 = yes)
Membership or attachment (dummy base: no membership or attachment to a group)					
ATTACH*	4,090	4	0.85	0.83	Attachment or commit. (0 = none; 3 = ultra)
PAYTV	4,090	2	0.54	0.50	Pay TV subscriber (1 = yes)
FANCLUB	4,090	2	0.10	0.30	Official fan club member (1 = yes)
ULTRA	4,090	2	0.06	0.24	Ultra group member (1 = yes)
Fan identity (factor variable) [two items: (i) identify with the club, (ii) keep up to date with my club]					
IDENTITY*	4,090	9	4.19	0.87	Fan identity (mean of 2 identification ite.)
IDENTIFICATION	4,090	5	3.89	1.15	Identification with the club (1‒5 sc.)
INFORMATION	4,090	5	4.49	0.83	Keep up to date with my club (1‒5 sc.)
FAVCLUB	4,090	2		0.26	Favorite club (1 = yes)
ACTIVEPART	4,090	5	2.80	1.53	Taking part in fan chants etc. (1‒5 sc.)
Attitudes toward commercial issues in football					
COMNECESS	3,813	5	3.75	1.10	Marketing of football necessary (1‒5 sc.)
MODERNFOOTB*	3,813	9	4.07	0.86	Issues of modern footb. (mean of 2 ite.)
COVERAGE	3,813	5	4.26	0.86	Increasing media coverage (1‒5 sc.)
SHOW	3,813	5	3.89	1.22	Show elements on matchdays (1‒5 sc.)
COSTS*	3,813	9	2.26	0.93	Issues of rising costs (mean of 2 ite.)
TRANSFER	3,813	5	2.23	1.05	Cost of player transfers (1‒5 sc.)
SALARIES	3,813	5	2.30	1.10	Level of player salaries (1‒5 sc.)
OBJECTIVES*	3,813	12	4.21	0.53	Issues of club objectives (mean of 3 ite.)
PROFIT	3,813	5	4.41	0.80	My club should aim for profits (1‒5 sc.)
WINNING	3,813	5	4.87	0.41	My club should aim for winning (1‒5 sc.)
STARS	3,813	5	3.36	1.07	My club should buy top stars (1‒5 sc.)
STADIUM*	3,813	17	3.91	0.72	Issues of stadiu. features (mean of 4 ite.)
PRICES	3,813	5	3.51	1.12	Ticket prices are reasonable (1‒5 sc.)
COMFORT	3,813	5	4.56	0.82	Comfort of new stadia (1‒5 sc.)
SPONSORS	3,813	5	4.16	0.95	Presence of sponsors in stadia (1‒5 sc.)
NAMING	3,813	5	3.39	1.44	Selling naming rights of stadia (1‒5 sc.)
Attitudes toward football governance					
REGULATION	3,299	5	1.96	1.16	Footb. needs regul. by authorities (1‒5 sc.)
SAFE	3,156	5	4.35	0.91	I feel safe going to a stadium (1‒5 sc.)
FANIMPORT*	3,156	13	4.55	0.62	Importance of Fans (mean of 3 ite.)
FANID	3,156	5	1.35	0.83	Introduct. of fan-ID Card is good (1‒5 sc.)
FANRIGHTS	3,156	5	4.30	0.97	Fans' rights are not suffic. consid. (1‒5 sc.)
FANSCULTURE	3,156	5	4.68	0.68	Fans' activities import. for Rus. fb. culture (1‒5 sc.)
CLUBSEXCLU	3,156	5	1.73	1.28	Agreem. exclus. clubs & nat. team (1‒5 sc.)
REFORM	3,156	5	2.59	1.24	Exclusion will lead to reforms (1‒5 sc.)
Attitudes toward club governance					
INVOLVEMENT*	3,299	9	3.39	0.63	Fan involvem. in club gov. (mean of 2 ite.)
APPROACH	3,299	5	4.60	0.69	Clubs should more approach fans (1‒5 sc.)
TRADITION	3,299	5	4.43	0.85	Clubs should strengthen tradition (1‒5 sc.)
RELATION	3,299	5	3.57	1.10	Good relation betw. fans & club (1‒5 sc.)
DECISION	3,299	5	3.18	1.21	Clubs may involve fans in decis. (1‒5 sc.)
NOINFLUENCE	3,299	5	4.33	0.99	My opinion doesn't influence club (1‒5 sc.)
CUSTOMER	3,299	5	3.63	1.16	Club owners/presid. treat fans as customers (1‒5 sc.)
GOODOWNER	3,299	5	2.47	1.12	Trust club owner/president do right thing (1‒5 sc.)
MONEYDEPEND	3,156	5	2.66	1.44	Club is too depending on state funding (1‒5 sc.)
Behavioral intentions					
LESSDEMAND*	3,556	13	2.09	0.84	Will demand less cl serv. (mean of 6 ite.)
LESSATT	3,556	5	2.43	1.17	Will attend less games of my club (1‒5 sc.)
NOMERCH	3,556	5	2.34	1.16	Won't buy club merchand. anym. (1‒5 sc.)
LESSCHEER	3,556	5	1.33	0.70	Will cheer less for the club (1‒5 sc.)
GIVEUPFAN	3,556	5	1.55	0.85	Will give up lived fan culture (1‒5 sc.)
LOWERDIV	3,556	5	1.52	0.90	Would go to a lower division club (1‒5 sc.)
TURNAWAY	3,556	5	1.50	0.90	Will quit football (1‒5 sc.)
Sociodemographic data					
GENDER	3,000	2	0.95	0.22	Gender (1 = male)
AGE	3,000	64	32.3	10.20	Age (years) [14; 82]
AGE2	3,000	64	1,148	772	Age (years) squared
EDUCATION	3,067	5	4.54	0.91	Education (1 = no certificate; 5 = master's degree)

The independent variables are categorized by groups of determinants of the dependent variables representing predominantly the item batteries in the questionnaire. Factor variables are marked by * and appear above the theoretically related item variables on discrete statements in the questionnaire, measured by five-point Likert scales throughout, resulting in five unique values each. Factor variables of attendance, attitudes, and behavioral intentions are computed by the means of the related two to six ordinal item variables, generating nine to 17 unique values in function of the distribution of distinct means. However, ATTACH and EDUCATION differ from the other factor variables because they are “quasi-ordinal”factor variables for nominal item variables. ATTACH: membership or attachment status, from no attachment to closer attachment, i.e., fan club membership with “ultra” representing the highest intensity of attachment. EDUCATION: from no certificate to master's degree at the highest.

In addition to item-level variables, Table 1 also presents factor variables, each aggregating two to six related items (e.g., STADIUM, encompassing variables related to stadium attendance). These are designed to capture broader constructs and enhance the robustness of regression analyses. Factor variables were computed from the corresponding items, with the exception of ATTACH, a quasi-ordinal measure representing degrees of fan attachment via formal membership. Attitudinal responses were measured using a five-point Likert scale (1 = strongly disagree to 5 = strongly agree), which has shown equivalency with longer scales in terms of reliability and validity (64).

In total, 47 variables were included in the exploratory analysis: 37 ordinal, six binary, and four metrics (including age and number of games attended). Regression analyses were conducted on four dependent variables: the ordinal TRUST_CLUB and TRUST_RUFB as well as binary versions, BINTRUST_CLUB and BINTRUST_RUFB, used in robustness checks. Ten factor variables and four sociodemographic variables were included in additional regressions for a more condensed model specification.

### 3.3 Survey development, sampling, and data collection

To ensure contextual relevance, the questionnaire—originally tested on German (65) and Chinese football fans (15)—was adapted for the Russian context. This included slight modifications to sociodemographic categories and stadium classifications. The translation into Russian was completed by a native Russian-speaking author. A professional back-translation was then performed by a Russian teacher proficient in German.

Given the sociopolitical context during the conflict in Ukraine and the need for reliable, valid, and diverse data, an online sampling method was deemed most appropriate. After a random pretest to assess clarity and internal consistency, the final survey was distributed between September 20 and October 24, 2022, via Telegram channels (see Table 2).

**Table 2:** Profile of telegram channels.

The name of the channel	Description	Numb. of channel members
Football factory	Channel of a Russian football blogger	60,305
Ball Production^a^	Channel about Russian and European football	52,981
Insights from Karp	Football journalist	30,536
Vadim Lukomski	Football observer	24,299
At Kuzmich's	Spartak Fans' Channel	14,600
Allanazarov | All about football in Russia	Channel about Russian football	12,900
Gorodnizkiy Football, money, game	Channel of a Russian football blogger	12,758
Judging with Igor Fedotov	Channel of the Russian football referee	12,196
No criminals	Channel about Russian football	12,145
Football Country	Channel about Russian football	11,191
No criminals	Channel about Russian football	12,145
Notes on site	Spartak Fans' Channel	10,941
Aloref	Channel of the Russian football referee	9,700
Resistance Mustache	Channel about the Russian Premier League	5,179
Football Topics|Lokalov	Football journalist	3,600
Ball Money|Football, Finance, Transfers	Channel about football statistics, finances and transfers	1,458
Temple of football	Community of Russian football fans	350

a This channel belongs to VK platform (instead of Telegram).

Adapted from Reichel et al. (16, p. 216). The number of channel members reflects figures at the time of the survey (October 2022) and may have changed subsequently.

Telegram functions as an international communication platform, serving over one billion users worldwide. The platform occupies a remarkable position within Russia's media and communication sphere. Despite previous institutional efforts to restrict access, the messenger remains one of the most widely used channels for news and public dialogue. For this reason, Telegram is often regarded as a comparatively autonomous digital space, where discussions on politically and socially sensitive issues may occur with fewer perceived constraints than on more heavily regulated media. This perception is reinforced by the presence of numerous unofficial channels, independent media outlets, and diverse discussion communities, which further contribute to its popularity as a venue for public dialogue, including debates related to sport.

The survey link was shared across a group of open, publicly available football-related channels. These channels varied in focus and audience, including those managed by sports journalists, referees, bloggers, and fan communities. While the survey sample is not statistically representative of all Russian football supporters, it reflects a diverse and engaged portion of the online audience. This approach allows us to capture a range of voices and perspectives visible in public discussions about Russian football on Telegram.

Of the 4,923 responses collected, 833 (17%) were incomplete or insufficiently (less than halfway) filled out, resulting in a final sample size of *N* = 4,090. The gender distribution was heavily skewed, with 95% male respondents and 5% female. Due to the wide regional distribution of participants and sample size, the dataset is considered approximately representative of active Russian football supporters who engage on social media (66). This is supported by the fact that 92% of respondents identified a favorite Russian professional club (FAVCLUB) and reported attending, on average, over five HOME matches per season. Fans attended fewer AWAY games, approximately three per season, which is consistent with the vast geographic distances between clubs in Russia.

High levels of fan IDENTITY were reported, with 76% (*M* = 4.19, *SD* = 0.87) expressing agreement or strong agreement with statements indicating attachment to their club. Moreover, 37% (*M* = 2.80, *SD* = 1.53) stated active participation in fan terraces, chants, choreographies, or group activities (ACTIVEPART). However, levels of formal organization were low: only 10% were members of official fan clubs (FANCLUB), 6% belonged to ULTRA groups, and only 10% held season tickets. In contrast, 54% subscribed to pay-TV (PAYTV) services to follow football.

The item batteries related to commercialization and fan behavior demonstrated strong internal reliability, with Cronbach's alpha values of *α* = .72 and *α* = .77, respectively. Other batteries, such as those concerning fan ID and international exclusion, were not intended to measure cohesive constructs. Summary statistics for all variables—including unique values, means, and standard deviations—are detailed in Table 1.

### 3.4 Data analysis strategy

The primary regression analysis examines factors influencing trust (“How much do you trust the following institutions with regard to the organization of football”) with focus on the two ordinal dependent variables TRUST_CLUB, which evaluates trust in the owner and the management of the favorite club, and TRUST_RUFB, which measures trust in Russian football governance, i.e., the Premier League, the National League and the association. Both are modeled using ordered logit regression techniques (67). For robustness checks, binary regressions were also conducted using the dichotomous variable BINTRUST_RUFB and BINTRUST_CLUB, where agreement with the commercialization statement is coded as 1 (agree/rather agree) and all other responses as 0. These binary models offer a more stringent test of hypothesized relationships.

All models were assessed for multicollinearity and heteroscedasticity, with no significant issues detected. The analyses were performed using Stata/SE 17.0. Additional methodological details, including access to the full survey instrument and codebook, are available upon request.

## 4 Results

### 4.1 Descriptive findings

The first descriptive finding indicates that Russian football fans have very limited trust in domestic football institutions. While trust in fan groups/supporters (TRUSTFANGR) is still the highest (62% with somewhat trust/trust completely, *M* = 3.65, *SD* = 1.02). By contrast, fewer than half of respondents trust the officials of the clubs, i.e., club owners (TRUSTOWNER) with 42% and *M* = 3.19 (*SD* = 1.04) and club management (TRUSTMANAG) with 47% and *M* = 3.28 (*SD* = 1.02). Even greater distrust is directed towards the institutions of the Russian league and football association. Accordingly, only 19% trust the Russian Premier League (TRUSTRPL, *M* = 2.46, *SD* = 1.08), 16% the Football National League, i.e., the second league division, (TRUSTFNL, *M* = 2.54, *SD* = 1.05), and 16% the Russian Football Association (TRUSTRFA, *M* = 2.30, *SD* = 1.09). Only about a quarter of respondents express trust in the international football associations UEFA (TRUSTUEFA, 25%, *M* = 2.47, *SD* = 1.24) and FIFA (TRUSTFIFA, 26%, *M* = 2.49, *SD* = 1.24).

In summary, trust in club governance (TRUST_CLUB, measured as the mean of TRUSTOWNER and TRUSTMANAG) is considerably higher (49%, *M* = 3.23, *SD* = 0.98) than in league and association governance (TRUST_RUFB, measured as the mean of TRUSTRPL, TRUSTFNL, and TRUSTRFA) (15%, *M* = 2.43, *SD* = 0.98). This outcome is reinforced by the low level of agreement (18%) with the statement that Russian fans “trust that the club owners/presidents will always do the right thing” (GOODOWNER, *M* = 2.47, *SD* = 1.12).

The skeptical attitude is further illustrated, for instance, by strong rejection of the introduction of the personalized fan ID required for purchasing tickets (FANID, *M* = 1.35, *SD* = 0.83), as well as by strong agreement with the statement that “the rights of fans are not sufficiently considered in Russian football” (FANRIGHTS, *M* = 4.30, *SD* = 0.97).

The critical stance toward institutions and the desire for less regulation is also reflected in the fact that 73% (rather) reject the idea that football needs REGULATION by the authorities. However, there is no contradiction when it comes to dependence on the state because only 30% [rather] agree that clubs are too dependent on state funding (MONEYDEPEND).

Although the attitude towards organizations and authorities in Russian football is generally quite critical, this does not mean that decisions made internationally are supported. Only 14% of respondents “agree to the exclusion of Russian clubs and the national team from international competitions” (CLUBSEXCLU, *M* = 1.73, *SD* = 1.28). Furthermore, only a minority (24%) of the fans surveyed “believe that exclusion from international competitions will lead to reforms in Russian football” (REFORM, *M* = 2.59, *SD* = 1.24).

### 4.2 Regression analyses results

Given the relatively little trust of Russian fans in football organizations, it is insightful to investigate the explaining factors of these attitudes. Further, it is also essential to generate evidence that proves the coherence and robustness of the regression findings.

The ordered logit regression on TRUST_CLUB and TRUST_RUFB in Model 3 is considered the key or “target model” as it captures the entirety of the data because it takes into account the full range of the five-point Likert scale. Conversely, Model 4 focuses on the factor variables as explanatory variables. Hence, information on the item level is lost; however, the major theoretical determinants are modelled to detect the coherence of the estimation on the item level. In other words, Model 4 illustrates consistency and robustness of Model 3's findings with its variants. Moreover, following the same estimation strategy, Models 1 and 2 were developed. Further on, we may largely focus on the results and insights given by the “target model” (Model 3).

#### 4.2.1 Fan trust in the club governance

A significant positive relationship between TRUST_CLUB (extent of trust in owner/management) and the explanatory item variable shows that agreement to the statement of the variable tends to contribute to the explanation of attitudes toward trust. This exploration is conducted as follows and is presented for TRUST_CLUB in Table 3 (Table 4).

**Table 3:** Regression results.

Independent variables	(1) Logit item model	(2) Logit factor model	(3) Ordered Logit item model	(4) Ordered Logit factor model
	BINTRUST_CLUB	BINTRUST_CLUB	TRUST_CLUB	TRUST_CLUB
ATTANDANCE		0.043***		0.023**
HOME	0.038***		0.018**	
SEASON	n.s.	n.s.	0.230*	0.231*
ULTRA	n.s.		−0.335**	
IDENTITY		n.s.		0.122**
IDENTIFICATION	−0.117*		n.s.	
INFORMATION	n.s.		−0.122**	
COVERAGE	0.183**		n.s.	
COSTS		0.246***		0.161***
TRANSFER	0.233***		0.141***	
STADIUM		0.245**		0.190***
PRICES	0.162**		0.147**	
SPONSORS	n.s.		0.116**	
SAFE	n.s.	n.s.	0.141***	0.159***
FANIMPORT		n.s.		−0.180***
FANID	0.228**		0.147***	
FANSCULTURE	0.214*		n.s.	
CLUBSEXCLU	−0.180***	−0.188***	−0.113***	−0.111***
REFORM	0.110**	0.103**	0.078**	0.086***
RELATION	0.773***	0.799***	0.736***	0.764***
TRADITION	−0.170*		−0.154***	
DECISION	−0.177***	−0.208*	−0.149***	−0.166**
MONEYDEPEND	−0.083*	n.s.	−0.070**	−0.048*
CUSTOMER	−0.272***	−0.255***	−0.260***	−0.259***
GOODOWNER	0.606***	0.606***	0.505***	0.515***
LESSDEMAND		0.170**		−0.231***
NOMERCH	−0.130*		−0.081*	
GIVEUPFAN	n.s.		−0.132**	
LOWERDIV	n.s.		0.169***	
TURNAWAY	−0.146*		−0.144**	
AGE	n.s.	n.s.	−0.049**	−0.057***
AGE2	n.s.	n.s.	0.001***	0.001***
*N*	2,849	2,849	2,849	2,849
McFadden's *R*^2^	0.277	0.263	0.196	0.188
Kelvey and Zavoi. *R*^2^	0.463	0.434		

All models were estimated in Stata 17.0. Only significant variables in at least one model are displayed. n.s. denotes non-significant. A blank cell means the omission of this variable in the model.

Significance levels: *p < 0.10, **p < 0.05, ***p < 0.01.

**Table 4:** Regression results.

Independent variables	(1) Logit item model	(2) Logit factor model	(3) Ordered Logit item model	(4) Ordered Logit factor model
	BINTRUST_RUFB	BINTRUST_RUFB	TRUST_RUFB	TRUST_RUFB
ATTANDANCE		n.s.		0.017**
HOME	n.s.		0.013*	
ATTACH		−0.116*		n.s.
PAYTV	0.212**		0.225***	
ULTRA	−0.653***		−0.296**	
IDENTIFICATION	−0.119**		−0.098**	
INFORMATION	0.183**		0.151***	
ACTIVEPART	n.s	−0.121***	n.s	−0.092***
COMNECESS	n.s	n.s	n.s	−0.117**
MODERNFOOTB		0.325***		0.249***
COVERAGE	n.s		0.102**	
SHOW	0.099**		0.061*	
COSTS		0.474***		0.377***
TRANSFER	0.342***		0.299***	
SALARIES	0.107*		n.s.	
PROFIT	−0.161**		n.s.	
STARS	0.117**		0.082**	
STADIUM		n.s.		0.160***
COMFORT	0.178**		0.143***	
NAMING	n.s.		0.057**	
REGULATION	0.152***	0.166***	0.140***	0.151***
FANIMPORT		−0.491***		−0.390***
FANID	0.188***		0.142***	
FANRIGHTS	−0.229***		−0.198***	
CLUBSEXCLU	−0.233***	−0.242***	−0.191***	−0.198***
REFORM	0.181***	0.175***	0.163***	0.165***
RELATION	0.175***	0.169*	0.185***	0.169*
TRADITION	−0.142**		−0.109**	
DECISION	n.s.	n.s.	−0.067**	n.s.
NOINFLUENCE	−0.134***	−0.129**	−0.119***	−0.112***
MONEYDEPEND	0.104***	0.102***	0.077***	0.080***
GOODOWNER	0.196***	0.207***	0.160***	0.165***
LESSDEMAND		−0.121*		−0.121**
TURNAWAY	−0.185**		−0.178***	
GENDER	−0.467**	−0.505**	−0.267**	−0.303**
AGE	−0.108***	−0.114***	−0.092***	−0.097***
AGE2	0.001***	0.001***	0.001***	0.001***
EDUCATION	−0.114*	−0.116*	n.s.	n.s.
* N*	2,849	2,849	2,849	2,849
* *McFadden's *R*^2^	0.174	0.158	0.116	0.106
* *Kelvey and Zavoi. *R*^2^	0.295	0.267		

All models were estimated in Stata 17.0. Only significant variables in at least one model are displayed. n.s. denotes non-significant. A blank cell means the omission of this variable in the model.

Significance levels: *p < 0.10, **p < 0.05, ***p < 0.01.

From this interpretation of the evidence, the following insights emerge: First, and not surprisingly, a general agreement on a good RELATION between fans and the (favorite) club, as well as the trust in the owners/presidents (GOODOWNER means “We can trust that the club owners/presidents will always do the right thing.”) strengthen trust in the club the most, each with the highest coefficient in the analysis. By contrast, trust in club management is lower when fans feel that owners/presidents treat them merely as CUSTOMERs. Agreement with the statement “Clubs may involve fans in decisions” also confirms lower trust in the club (DECISION).

Second, agreement with certain commercial aspects, such as high TRANSFER fees or reasonable ticket PRICES, strengthens confidence in club officials. On the other hand, a critical attitude towards increasing commercialization, expressed by the statement that football matches and products will be less in demand in the future (factor LESSDEMAND), reflects greater mistrust.

Third, a generally (sports) politically reflective attitude limits trust. This is evident both in the rejection of the introduction of the FANID, but also in the approval of Russian clubs and the national team being excluded from international competitions (CLUBSEXCLU). Conversely, the feeling of safety (SAFE) in the stadium strengthens trust in the club officials.

Regarding the sociodemographic results, there is at least weak evidence that older respondents tend to trust less. Unlike studies on European football, other socio-demographic characteristics such as gender and education do not show any significance in the regression analyses. Nevertheless, similar results can be clearly derived from the descriptive findings. In terms of GENDER, women (*M* = 3.55, *SD* = 1.01) trust club management slightly more than men (*M* = 3.34, *SD* = 0.99), and skepticism increases continuously with higher levels of EDUCATION.

#### 4.2.2 Fan trust in the Russian football governance

As before, a significant positive relationship between TRUST_RUFB (level of trust in the Russian Premier League, Football National League and Russian Football Association) and the explanatory variable indicates that agreement with the statement of the variable tends to contribute to explaining attitudes towards trust (Table 5).

**Table 5:** Trust in governing football institutions: European total, six countries and Russia (measured using a Likert scale (1 = strongly disagree to 5 = strongly agree).

	Means by country (SD)							
Q. How much do you tend to trust the following institutions?	European total	France	Germany	Poland	Spain	Turkey	UK	Russia
Fan groups/supporters'organizations	3.48 (1.05)	3.07	3.68	3.91	3.49	3.20	3.73	3.65 (1.02)
Club management	3.23 (1.05)	3.43	3.51	3.66	2.36	2.93	3.41	3.28 (1.02)
UEFA	2.60 (1.19)	3.00	2.36	3.45	2.38	2.62	2.32	2.47 (1.24)
Professional Football League	2.55 (1.16)	2.53	3.07	3.25	1.96	1.79	2.76	2.46 (1.08)
National Football Federation	2.49 (1.20)	2.59	3.03	3.13	2.08	1.56	2.62	2.30 (1.09)
FIFA	2.31 (1.22)	2.56	1.85	3.40	2.28	2.74	1.87	2.49 (1.24)

European data from Garcia and Llopis-Goig (14); Russian data from authors' survey.

The following key findings can be derived: First, and as studies on European football have shown, ULTRAs—and, to a lesser extent, (active) fans (ACTIVEPART) who identify (IDENTIFICATION) strongly with their favorite club—are very critical of league and association governance and therefore have very little trust in these organizations. Contrary, fans who regularly keep up to date with the club's activities (INFORMATION) and assess the RELATIONship between fans and the (favorite) club, as well as the relationship of trust with the owners/presidents (GOODOWNER) positively not only maintain good relations with their favorite club but also express greater trust in the leagues and associations in Russian football.

Second, agreement with certain aspects of modern football (MODERNFOOTB), including increasing media COVERAGE and eventization as part of the match (SHOW), issues of rising COSTS, such as high TRANSFER fees, and aspects of a modern STADIUM, like the increasing COMFORT, can be seen as an explanation for greater trust in Russian football governance. Fans who may want to TURNAWAY from professional football due to increasing commercialization show significantly lower trust.

Third, if respondents critically evaluate changes and consider fans' rights to be important, their trust in Russian football governance declines (factor FANIMPORT). Specifically, this skepticism is evident in the introduction of the FANID and the opinion that fans' rights are not sufficiently considered in Russian football (FANRIGHTS). From a (sports) policy perspective, approval of the exclusion of Russian teams from international competitions also leads to significantly lower trust. On the other hand, the belief that this exclusion will lead to REFORMs in Russian football and a positive attitude towards the authorities (REGULATION: “Football needs regulation by the authorities.”) increases trust in Russian football governance.

Regarding the sociodemographic results, there are clear similarities with European football. Regarding GENDER, women trust club management significantly more than men and trust decreases slightly with increasing AGE in a highly significant manner. Finally, there is a tendency for trust in club management to decline with increasing EDUCATION.

## 5 Discussion and conclusion

This article examines the attitudes and trust of Russian football fans towards football governing bodies using quantitative research design. The findings of this study indicate that trust and mistrust remain as a key driver in the relationship between football fans and sport governing institutions. The broad pattern reflects data previously obtained from European countries (14, 40). Specifically, the following findings can be summarized:

1. Overall, the fans surveyed show little trust in sport organizations and institutions in Russian football. Nevertheless, the greatest trust is placed in supporter groups, while they show only moderate trust in club management. However, they exhibit notably low trust in national (football league and association) and international football governing bodies (UEFA and FIFA). This trend confirms a general pattern that trust declines as the institutional distance between fans and decision-making governing bodies increases. These findings are in line with results in European football by Garcia and Llopis-Goig (14).
2. Trust in the management of a favorite club is shaped by having a good relationship with the club and a positive attitude toward the owner. A positive attitude toward commercial aspects of football, such as the amount of transfer fees or management decisions like the appropriate level of ticket prices, strengthens trust. Conversely, one of the key factors contributing to mistrust is the introduction of fan ID, illustrating how policies aimed at increasing regulation can undermine legitimacy of governing institutions and distance fans, when presented as a security measure. Moreover, declining attendance, boycotts and ongoing opposition to the fan ID highlight the potential consequences for development of Russian football. Compromise, protecting the rights of supporters and their meaningful participation in decision-making processes at all levels of football governance are crucial to fostering and maintaining legitimacy.
3. Trust in Russian football governance reveals that the level of trust depends on the type of fans. Ultras and active fans are highly skeptical of local football organizations, thus confirming their critical attitude towards the institutional governance of contemporary football. However, other fans who regularly follow the club's activities perceive their relationship with the club more positively and express a high level of trust in both the club and the league. Therefore, we observe a reverse trend, in which a positive perception of club governance appears to correlate with increasing trust in supra-club governance structures. Additionally, confidence of Russian fans decreases with age and higher educational attainment, while women are more trusting. These general tendencies are consistent with the results of a European study (14). The other trends were only partially confirmed and were not considered sufficiently significant in the regression analysis.

### 5.1 Theoretical and practical contributions

The contribution of the study lies in three areas. First, it provides systematic evidence and extend debates on trust in a non-Western football context (beyond the European Big-Five leagues). The findings on Russian football are particularly significant for debates among sports economists regarding spectatorship. The results highlight the importance of regional and national characteristics in shaping consumer attitudes, behaviors, and concerns. In terms of trust, Russian fans display similar attitudes and concerns to fans in Europe. This seems surprising, as previous fan studies concentrating on other relevant governance issues in professional football, such as (increasing) commercialization, reveal notable differences compared to European context (16). Moreover, the evidence also emphasizes that trust in football governance depends not only on performance and commercialization but also on broader political and institutional context. Therefore, it is worth exploring the data in greater depth and providing a better insight into the culture of consumption in sport, not only in different countries, but also in different regions within those countries. Overall, comparative empirical approaches in sports economics research could be developed more intensively, as culture and institutions are vital and are frequently overlooked in economic models of sport.

Second, the Russian example highlights the importance of the political or more broadly governance system in which a sport operates, along with its historical traditions. Accordingly, the institutional setting of the sports industry and its market mechanisms can be seen as a significant factor to integrate into theoretical demand models in sports economics. A promising path forward may involve combining insights from political economy and institutional economics with established neoclassical demand models.

Third, in response to the recent military conflict in Ukraine, numerous countries and international sports organizations imposed sanctions on Russia's economy and sporting sector, with the intention of encouraging public pressure for change. Regarding football supporters, our study reveals a critical stance among Russian football fans toward governing bodies in the sport, though the evidence does not suggest that these measures have had a substantial impact on the broader football system.

As a practical implication, the study's results once again emphasize that club management should acknowledge and respect fans' psychological ownership (68, 69) rather than viewing them merely as customers, if they wish to strengthen fans' trust. Fans who demand involvement in club decisions tend to exhibit lower levels of trust, as they feel excluded from meaningful participation. Offering genuine opportunities for engagement can potentially enhance their trust, particularly when they perceive their input as having a real impact. However, implementing such measures would require a broader transformation within Russian sports governance.

### 5.2 Limitations and future research

This study has certain limitations. First, the survey's reach was restricted, as it was promoted only through two digital social platforms. Recent studies highlight systematic sociodemographic differences between online survey participants and the general population. Respondents in online surveys are typically younger, more highly educated, and more likely to live in urban areas, whereas older, less-educated, and rural individuals are underrepresented. A slight underrepresentation of women is also observed. These disparities primarily result from coverage and self-selection bias, rather than from pure survey mode effects (70, 71). Second, the study coincided with a challenging political climate in the country. This was evident when several administrators of large Telegram channels declined to share information about the survey, some for political reasons, but more commonly because they only agreed to publish content for a fee. Nonetheless, despite these hurdles, the survey achieved wide coverage: fans from 81 regions participated, representing an impressive 95% of all regions in Russia. It should also be noted that Telegram, a platform that many Russian users regard as maintaining a relatively high degree of autonomy. Therefore, this perception may have influenced respondents to express somewhat more critical or independent perspectives toward local football organizations.

Another potential limitation is that not all types of fan groups, for instance football hooligans, were represented in the survey. In fact, one of the largest online communities associated with this group declined to share information about the study, citing skepticism and mistrust toward the academic community. The findings reveal complex and often contradictory attitudes among fans regarding their trust in sports governing bodies. To gain a deeper understanding of the relationship between fans as consumers and sports organizations as providers, further research is required, particularly beyond the Big-Five European leagues and in non-Western contexts with diverse political and institutional settings.

## Data Availability

The original contributions presented in the study are included in the article/Supplementary Material, further inquiries can be directed to the corresponding authors.
